# Impact of metastable defect structures on carrier recombination in solar cells†

**DOI:** 10.1039/d2fd00043a

**Published:** 2022-04-11

**Authors:** Seán R. Kavanagh, David O. Scanlon, Aron Walsh, Christoph Freysoldt

**Affiliations:** Department of Chemistry & Thomas Young Centre, University College London 20 Gordon Street London WC1H 0AJ UK sean.kavanagh.19@ucl.ac.uk; Department of Materials & Thomas Young Centre, Imperial College London Exhibition Road London SW7 2AZ UK; Department of Materials Science and Engineering, Yonsei University Seoul 03722 Republic of Korea; Max-Planck-Institut für Eisenforschung GmbH Max-Planck-Str. 1 40237 Düsseldorf Germany

## Abstract

The efficiency of a solar cell is often limited by electron–hole recombination mediated by defect states within the band gap of the photovoltaic (PV) semiconductor. The Shockley–Read–Hall (SRH) model considers a static trap that can successively capture electrons and holes. In reality however, true trap levels vary with both the defect charge state and local structure. Here we consider the role of metastable structural configurations in capturing electrons and holes, taking the tellurium interstitial in CdTe as an illustrative example. Consideration of the defect dynamics, and symmetry-breaking, changes the qualitative behaviour and activates new pathways for carrier capture. Our results reveal the potential importance of metastable defect structures in non-radiative recombination, in particular for semiconductors with anharmonic/ionic–covalent bonding, multinary compositions, low crystal symmetries or highly-mobile defects.

## Abstract 2

Metastable defect structures can activate novel pathways for electron–hole recombination in semiconductors – particularly for inorganic compounds with anharmonic/mixed bonding, multinary composition, low symmetry and/or highly-mobile defects.

## Graphical abstract

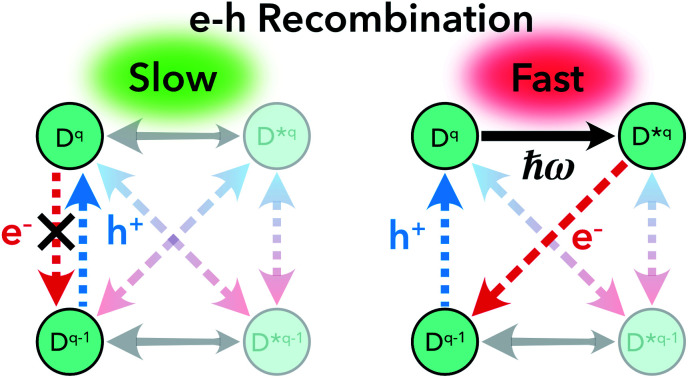

## Introduction

The search for new thin-film photovoltaic (PV) materials must balance advantageous properties (such as high absorbance, a band gap matching the targeted light spectrum, easy separation of electrons and holes, *etc.*) with the susceptibility to performance-limiting loss mechanisms. Among these, defect-mediated non-radiative recombination represents the dominant loss mechanism in emerging inorganic PV technologies.^1,2^ Identifying the ‘killer’ defect traps and hence potential synthesis and processing strategies to avoid their effects is crucial to mitigating losses and achieving high performance. For instance, the major efficiency improvements of the last 5 years for single-junction perovskite solar cells can be directly attributed to effective trap management.^3^

Point defects, such as vacancies, interstitials and antisites, facilitate carrier recombination by breaking the periodicity of the solid and thus introducing new electronic states within the semiconducting band gap. By successively capturing electrons and holes through non-radiative (vibrational) processes, these defect levels allow excited charge carriers to recombine across the gap, reducing the open-circuit voltage in solar cells and the quantum efficiencies of light-emitting diodes for example.^1^ Recent advances in both theory and computation have rendered possible the explicit prediction of defect recombination activity,^4–9^ allowing for the *ab initio* identification of such ‘killer’ traps. The synergistic combination of theoretical methods and experimental characterisation has allowed for rapid improvements in photoconversion efficiencies for emerging inorganic PV materials.^1,10^

Point defects can exhibit complex potential energy surfaces (PESs), with multiple local minima.^9,11,13–15^ As such, their atomic structures often do not reside solely in their global equilibrium configuration, but may access alternative metastable structures *via* thermal, photo or electronic conversion. The possibility of structural transformation at defect sites has several potential impacts for technological applications—for example, low energy barriers between structures could lead to coherence corruption and a breakdown in performance for defects used in quantum computing and single-photon emission. In particular, carrier capture rates at defects are sensitively dependent on the atomic structure, varying by over 15 orders of magnitude with different geometries for the same nominal defect species,^9,16^ and so the presence of these alternative configurations can introduce alternative pathways which significantly alter the overall recombination kinetics. Indeed, this phenomenon has recently been reported in the literature, with metastable defects accelerating the electron–hole recombination process and transforming benign defect centres into harmful traps.^9,16–18^

A complete analysis of the possible mechanisms and resulting impact of metastable configurations on non-radiative recombination in semiconductors is lacking. In this work, we first provide a general analysis of the potential recombination pathways introduced by metastable defect structures. We discuss the conditions for this behaviour to occur, the anticipated trends and the factors governing the consequences for material properties. We highlight tellurium interstitials in CdTe as illustrative exemplars of the potential complexity and importance of metastable configurations to carrier recombination, exhibiting a novel thermal-excitation recombination cycle which has not previously been reported in the literature, to the knowledge of the authors. Finally, we discuss the important implications of these findings to the broader field of photovoltaic materials research.

## Metastable defect dynamics

**Fig. 1:**
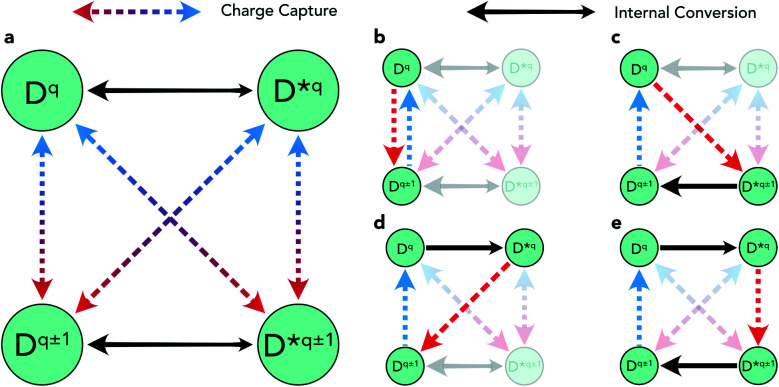
(a) Graph network illustration of the potential structural transitions involving charge capture (red/blue) or internal conversion (black) for a defect *D* in charge state *q*. A single metastable configuration D*^q^ is assumed to be accessible for each charge state. (b) Pathway for a typical 2-step Shockley–Read–Hall recombination process. (c) Pathway for a 3-step recombination process involving *capture into* a metastable defect structure, followed by internal relaxation and charge capture back to the initial state. (d) Pathway for a 3-step recombination process involving *internal excitation* to a metastable structure, followed by electron and hole charge capture. (e) Pathway for a 4-step recombination process involving *internal excitation* to a metastable structure (D*^q^), charge capture to the *q* ± 1 metastable structure (D*^q±1^), *internal relaxation* and charge capture back to the initial configuration (*D*^*q*^)

There are several mechanisms by which the presence of metastable structures can influence the overall defect behaviour in solids. Regarding non-radiative carrier recombination, the structural transition pathways introduced by metastable configurations on the defect potential energy surface can act to accelerate or retard this process, or may not affect it at all. Fig. 1 illustrates these possible transitions using a simplified graph network for the case of a single metastable configuration D* that is both accessible and significantly influential on the overall capture behaviour.

The impact of these metastable defects depends on the competing charge capture rates (dashed red/blue arrows), the relative energies *of* and transition barriers *between* ground and metastable configurations (*i.e.* their accessibility) (solid black arrows) and the defect energy level position in the host band gap (eqn (1)). Each of the charge capture transitions can be fast or slow, with no *necessary* correlation between opposing directions. That said, the recurrence of harmonic PESs means that often a fast electron capture rate implies slow hole capture in the reverse direction, and *vice versa*.^1,9,19^ This is typically the result of the defect level lying close to the valence or conduction band, often yielding strong/weak interaction with the near/distant band edge and thus fast/slow carrier capture. This is the intuitive rationale behind the expectation that deep midgap defect levels will act as efficient recombination centres.^1,16,19,20^ In many cases however, this trend is violated as a result of anharmonicity in the defect PES, often arising due to symmetry-breaking in the defect structure.^7,9,21^ The prevalence of this phenomenon in semiconductors is becoming increasingly better understood, owing to advances in experimental characterisation and computational modelling techniques.^1,9,11,22–24^ On the other hand, there is a clear and definite correlation between the opposing internal conversion directions, indicated by the relative sizing of the black arrows in Fig. 1. The transition energy barrier from ground to higher energy metastable configuration must be larger than the reverse, hence *k*_*D*→D*_ < *k*_D*→*D*_ where *k* is the transition rate constant (*i.e.* it is always easier to thermally relax than to excite).

Transitions to higher charge states (*q* ± 2) have not been included here as the behaviour is mostly equivalent and, regardless, for recombination to occur, a closed path must be established (as in Fig. 1b–e), returning the defect to the initial configuration. That said, two ways in which *q* ± 2 defects *could* influence the overall recombination rate here is (1) by introducing rapid capture into, but slow capture out of, these extremal charge states, thus acting to inhibit recombination as defects get ‘stuck’ in inert charge states,^1,16,21^ or (2) the rare scenario where *D*^*q*±2^ introduces an alternative low-barrier pathway between *D*^*q*±1^ and D*^q±1^ which does not exist otherwise, potentially accelerating the recombination process (*i.e.* introducing an additional path along the lower rim of the graph network in Fig. 1a).

Fig. 1c serves as an illustrative example of the potential acceleration or deceleration of the recombination cycle by metastable structures, with the rate of the internal relaxation (black arrow) from D*^q±1^ to *D*^*q*±1^ being the deciding factor. Assuming rapid capture transitions for *q* → q* ± 1 (red) and *q* ± 1 → *q* (blue), a fast internal relaxation means that D*^q±1^ acts as a reaction intermediate, introducing a rapid pathway for the recombination cycle to proceed. If, however, internal conversion is extremely slow and D*^q±1^ does not readily relax to *D*^*q*±1^, several alternative pathways may emerge. The long-lived metastable configuration could undergo charge capture and return back to the original *D*^*q*^, completing the e–h recombination cycle without involving *D*^*q*±1^. Such behaviour would increase/decrease the overall recombination rate if the rate-limiting step for *D*^*q*^ ⇔ D*^q±1^ was faster/slower than that of *D*^*q*^ ⇔ *D*^*q*±1^. Instead, if the defect level is sufficiently close to a band edge D*^q±1^ could readily emit the captured charge, acting as a shallow defect level with no effect on the recombination rate. Alternatively, if D*^q±1^ has both slow internal relaxation *and* slow charge capture, defects could become ‘stuck’ in this inert state and in fact greatly diminish the overall recombination activity.

In the above discussion, only non-radiative (phonon emission) pathways are considered. Under certain conditions, both charge capture and internal conversion may also proceed radiatively through photon emission. A detailed consideration of such behaviour is beyond the scope of this report, though we note that it is non-radiative capture which dominates efficiency losses in PV semiconductors.^1,4,25^

### Capture *into* metastable defects

In recent years, several papers have highlighted the decisive role that metastable defects can play in charge-carrier capture kinetics.^9,16–18^ In each of these studies, it is the situation described in Fig. 1c that is reported, where the defect *D*^*q*^ captures an electron or hole by transforming to a metastable configuration of the *q* ± 1 defect (D*^q±1^). This metastable species then relaxes to the ground-state *D*^*q*±1^ through an internal conversion reaction, whether a spin-flip de-excitation^16^ or (more commonly) structural/vibrational relaxation,^17,18^ or both.^9^ In each case, this alternative pathway is found to proceed more rapidly than the standard *D*^*q*^ ⇔ *D*^*q*±1^ charge capture process (Fig. 1b), thus enhancing the overall recombination rate and reconciling theoretical recombination models with experimental observations.

The question of whether one should expect this to typically be the case, where the presence of a low-lying metastable structure D*^q±1^*increases* the recombination rate, is still up for discussion. The reporting of this phenomenon in the literature is undoubtedly biased, likely only to be discussed when the metastable defect accelerates recombination and ignored otherwise, thus discouraging any general conclusions based on the prevalence of these observations.

**Fig. 2:**
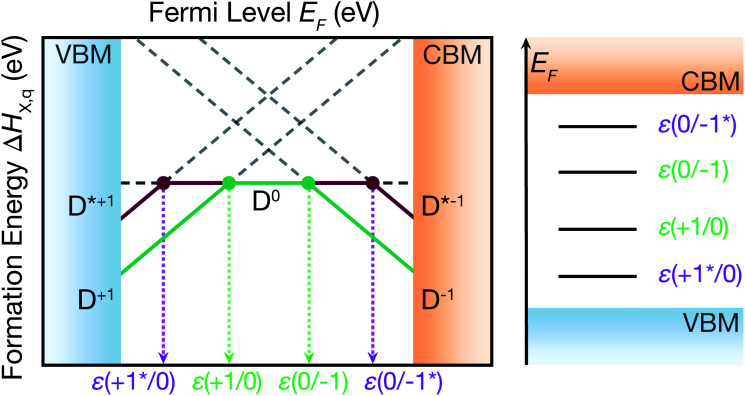
Charge transition level positions for ground-state *ε*(*q*/*q* ± 1) and ground-state ⇔ metastable configurations *ε*(*q*/q* ± 1), on a defect formation energy diagram (left) and a vertical energy level diagram (right).

The shift in defect energy levels when metastable defects are involved (depicted in Fig. 2), does however permit some general expectations for the relative capture rates. As mentioned above, much of the electronic behaviour of point defects is dictated by the position of their energy levels in the semiconducting band gap. These in-gap levels are given by their ‘thermodynamic charge transition level’ (TL) positions *ε*(*q*_1_/*q*_2_), which are the Fermi level positions where the equilibrium charge state switches between *q*_1_ and *q*_2_, given by:1
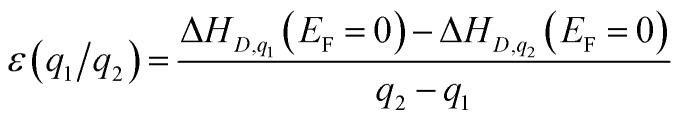
where Δ*H*_*D*,*q*_(*E*_F_ = 0) is the formation energy of defect *D* with charge *q* when the Fermi level is at the zero-reference point (the VBM, by convention). Taking *q*_1_ = *q*, *q*_2_ = *q* ± 1, and denoting the energy of the metastable defect Δ*H*_D*,*q*±1_ as:

2 Δ*H*_D*,*q*±1_ = Δ*H*_*D*,*q*±1_ + Δ*E*

where Δ*E* is the energy of the metastable defect relative to the ground-state structure, we can then substitute into eqn (1) and rearrange (full derivation in Section S1.1†) to obtain:

3 *ε*(*q*/q* ± 1) = *ε*(*q*/*q* ± 1) ∓ Δ*E*

4 e^−^ capture ⇒ *ε*(*q*/q* − 1) = *ε*(*q*/*q* − 1) + Δ*E*

5 h^+^ capture ⇒ *ε*(*q*/q* + 1) = *ε*(*q*/*q* + 1) − Δ*E*

Thus we witness that for charge capture *into* a metastable structure, *D*^*q*^ → D*^q±1^, the transition level will move an energy Δ*E closer to* the corresponding band edge (*i.e.* to higher energy in the band gap for electron capture or to lower energy for hole capture), assuming a transition level *ε*(*q*/*q* ± 1) initially located within the band gap. Conversely, this shift in TL position also means the TL will be located *further* from the relevant band edge for the D*^q±1^ → *D*^*q*^ transition (capture *from* the metastable defect). For example, the *ε*(0/−1*) transition level is located closer to the CBM, from which electrons are captured by *D*^0^ to form D*^−1^, than *ε*(0/−1), but further from the VBM, from which D*^−1^ could capture holes to complete the recombination cycle. Under the conventional rationale of capture rates being exponentially dependent on the separation of the defect level from the band edge,^1,9,19,20^ this implies a faster capture into the metastable configuration (*D*^*q*^ → D*^q±1^) than the ground-state process (*D*^*q*^ → *D*^*q*±1^), but a slower capture rate in the opposite direction (D*^q±1^ → *D*^*q*^). As discussed above, if D*^q±1^ can swiftly relax to *D*^*q*±1^, the situation depicted in Fig. 1c emerges. Here the ‘best of both’ is obtained, with electron capture proceeding *via* the TL nearest the conduction band and hole capture *via* the TL nearest the valence band, likely expediting the recombination process as observed in ref. 9 and 16–18. Crucially, the introduction of the internal conversion to the recombination kinetics means that the timescale of the D*^q±1^ → *D*^*q*±1^ transition, relative to the slowest charge capture process, will dictate whether the metastable defect accelerates or retards the overall recombination rate. Given typical effective vibrational (attempt) frequencies of ∼0.5–10 THz,^9,18,26^ internal conversion is unlikely to be the rate-limiting step in a fast recombination cycle if the transition energy barrier Δ*E* is less than 0.2–0.4 eV (Section S3.1†).

Another important consideration is that for the metastable configuration to enter the recombination process, the transition level *ε*(*q*/q* ± 1) must lie within the band gap formed between the valence band maximum (VBM) and conduction band minimum (CBM):

6 *E*_VBM_ < *ε*(*q*/q* ± 1) < *E*_CBM_

Using eqn (3) to substitute for *ε*(*q*/q* ± 1):

7 *E*_VBM_ < *ε*(*q*/*q* ± 1) ∓ Δ*E* < *E*_CBM_

Thus for electron capture we have:

8 *ε*(*q*/*q* − 1) + Δ*E* < *E*_CBM_

9 e^−^ capture ⇒ Δ*E* < *E*_CBM_ − *ε*(*q*/*q* − 1)

and for hole capture:

10 *E*_VBM_ < *ε*(*q*/*q* + 1) − Δ*E*

11 h^+^ capture ⇒ Δ*E* < *ε*(*q*/*q* + 1) − *E*_VBM_

Hence, the energy window between the ground-state transition level *ε*(*q*/*q* ± 1) and the corresponding band edge sets an upper limit on the relative energy of the metastable structure, if it is to yield a transition level within the band gap and potentially affect carrier recombination (eqn (9) and (11)). For instance, for D*^−1^ in Fig. 2 to introduce a transition level within the band gap, the energy of D*^−1^ relative to *D*^−1^ must be less than the energy separation of *ε*(0/−1) and the CBM.

As an example, in CdTe, there are 6 intrinsic defects (two vacancies, antisites and interstitials each), which range in charge from 0 to either −2 or +2 for a Fermi level within the band gap (except Te_Cd_ which ranges from −2 to +2).^27^ These 20 defect species yield 14 in-gap levels involving only ground-state structures. With our recently-published defect structure-searching technique (see Methods),^11,12^ we further identify 14 *metastable* structures for these charge states, generating 32 additional charge transition levels. Only 12 of these structures are sufficiently low energy to yield at least one transition level within the band gap, including those for Te_i_ discussed here and V_Cd_ discussed in ref. 9, yielding 18 transition levels involving metastable structures in the CdTe band gap. Of course, the presence of low-energy metastable defect structures in a given material will depend on several factors, including the bulk crystal symmetry, chemical bonding, and band gap, amongst others.

We note that Fig. 2 also serves as an illustrative example of the fluctuations in charge transition level positions that temperature and strong electron–phonon coupling would engender, yielding a distribution of energy levels rather than a static trap. Such behaviour could then influence the non-radiative recombination process in a similar fashion to the impact of metastability discussed here, with non-linear temperature dependence in capture cross-sections—an important consideration when interpreting the results of spectroscopic defect measurements.^28^

### Capture *from* metastable defects

As illustrated by Fig. 1d and e, metastable configurations can also enter the recombination cycle through an initial thermal or optical excitation, *prior* to charge capture. Again, the metastable defect D*^q^ could have a slower or faster capture rate than the corresponding ground-state configuration *D*^*q*^. In the case of faster capture (*k*_D*→*D*^*q*±1^_ > *k*_*D*→*D*^*q*±1^_), the metastable configuration acts as an accelerating reaction intermediate to electron–hole recombination through the path *D*^*q*^ → D*^q^ → *D*^*q*±1^ → *D*^*q*^ (Fig. 1d). This mechanism is similar to the *D*^*q*^ → D*^q±1^ → *D*^*q*±1^ → *D*^*q*^ route (Fig. 1c) discussed in the previous section, though with some crucial differences in the governing kinetics (now involving an internal excitation, rather than relaxation). If accessible metastable configurations exist for both charge states, then recombination could also proceed through the pathway shown in Fig. 1e, with the charge capture transition occurring between the metastable defects D*^q^ → D*^q±1^, prior to de-excitation and charge capture back to *D*^*q*^. While certainly possible, to our knowledge a defect centre exhibiting this behaviour has not been previously reported in the literature, which may be attributed to the recency of advancements in atomistic modelling of non-radiative carrier recombination^4–6,8,29^ and our understanding of the importance of metastable structures in these processes.^9,16–18^ Interestingly, we note that an analogous hot-electron capture mechanism was however invoked to explain the puzzling experimental observations of charge capture behaviour for negative-*U* DX centres in Al_*x*_Ga_1−*x*_As in the 1980s.^30^

#### Tellurium interstitial (Te_i_) in CdTe: structures

**Fig. 3:**
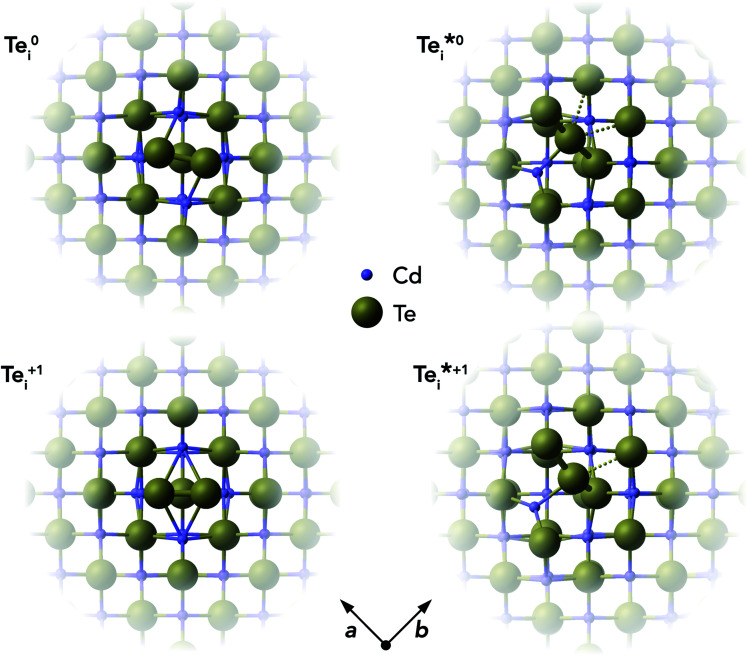
Atomic structures of ground-state (Te^0/+1^_i_) and metastable 
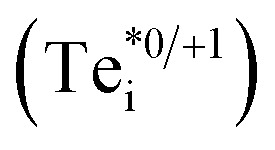
 tellurium interstitials in CdTe, looking down the [001] axis. Atoms sized according to their formal ionic radii.^31^ Te anions are shown in gold and Cd cations in purple.

**Fig. 4:**
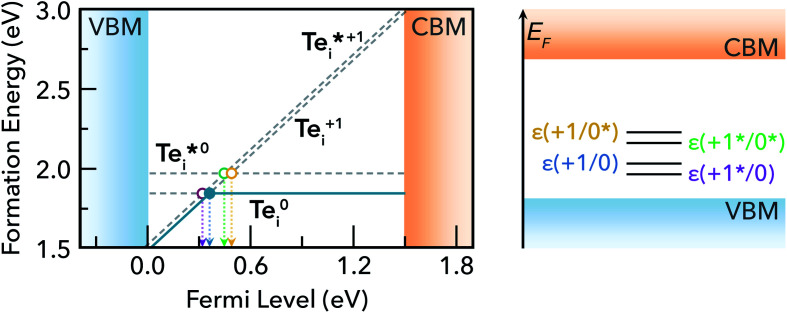
(Left) Defect formation energy diagram for the neutral and positively charged states of Te_i_ in CdTe, under Te-rich conditions (*μ*_Te_ = 0). The equilibrium charge state is shown in solid blue, and higher energy states in dashed grey. (Right) Vertical energy band diagram showing the Te_i_ (+^(^*^)^/0^(^*^)^) charge transition levels in CdTe.

In our calculations of point defects in CdTe, we found the low-energy tellurium interstitial (Te_i_) to be an exemplar of this charge capture phenomenon. The individual defect structures and corresponding transition level diagram for Te_i_ in CdTe are shown in Fig. 3 and 4 respectively.

We find that Te_i_ has two low-energy structural arrangements for both the neutral and positively charged interstitial, resulting in four potential charge transitions (*i.e.* intersection points on the transition level diagram in Fig. 4). In both the neutral and positively-charged ground-states, the interstitial Te_i_ displaces a host Te atom to form a split-interstitial Te–Te dimer (Fig. 3), as first noted by Du *et al.*^32^ For Te^+1^_i_, the dimer bond is directed along the 〈110〉 crystal direction, adopting a *C*_2v_ point group symmetry. For Te^0^_i_, the dimer bond is twisted slightly (12°) about the [001] axis, thus reducing to *C*_2_ point group symmetry with the removal of the {110} mirror plane.

A standard *ab initio* geometry relaxation from the high-symmetry tetrahedral interstitial coordination does not find the neutral ground-state Te^0​^_i_, but rather converges to a higher-energy structure 
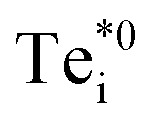
 with a formation energy only 127 meV higher than the split-interstitial dimer ground-state (Fig. 4). This configuration exhibits two short (2.90 Å) and two long (3.86 Å) Te_i_–Te distances, and an apical Te_i_–Cd bond (2.66 Å) yielding *C*_2v_ point symmetry (Fig. 3). In fact, this structure is a saddle point on the PES, representing the transition state of the Te_i_ interstitial diffusion mechanism (*i.e.* hopping of the split-interstitial dimer).^26,33,34^ We thus determine a diffusion barrier of 0.13 eV for Te^0^_i_ using the HSE(34.5%) + SOC functional (Fig. S2†), similar to the literature value of 0.09 eV calculated using both GGA (PBE) and hybrid (HSE06) DFT.^26,33^ A similar metastable structure exists for the positively-charged interstitial 
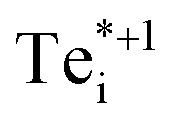
, where the partial occupancy of the defect level induces a Jahn–Teller splitting of the two long Te_i_–Te bonds, thus reducing to *C*_s_ point symmetry (Fig. 3). Here, the metastable configuration 
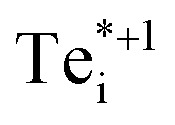
 is only 36 meV higher in energy than the ground-state Te^+1^_i_, now exhibiting a local stability window of 40 meV (Fig. S2†).

#### Te_i_ in CdTe: site degeneracies

Beyond the introduction of additional potential recombination pathways, the varying symmetries of the Te_i_ configurations yields differing site degeneracies for these structures. Site degeneracy is inversely proportional to the number of symmetry operations, and so low-symmetry, distorted defect structures can possess much larger degeneracies than high-symmetry defects. Given the linear relationship between defect concentration and configurational degeneracy *N* ∝ *g*, these effects are not insignificant, and can alter the defect concentrations by over an order of magnitude in certain cases. For Te^+1^_i_, there are six equivalent 〈110〉 directions in which the cubic structure can distort to form the *C*_2v_ symmetry split-interstitial (with the Te dimer bond oriented along one of the 6 〈110〉 directions). In combination with a spin degeneracy of 2 arising from the unpaired electron, the total configurational degeneracy is thus *g*_+1_ = 12. There are two equivalent clockwise/anti-clockwise rotations about the [001] axis which give the neutral Te^0^_i_ twisted dimer (*C*_2_ symmetry) (Fig. 3), and no unpaired electrons, which respectively double and half the degeneracy thus giving *g*_0_ = *g*_+1_ = 12. For the saddle-point neutral defect 
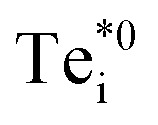
, the *C*_2v_ structure involves two Te neighbours moving closer to the interstitial atom, and two moving away (Fig. 3), giving a configurational degeneracy of 
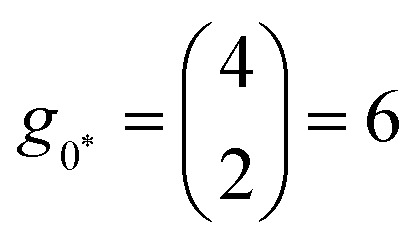
. The splitting of the two long Te–Te bonds in the *C*_s_
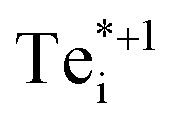
 structure (Fig. 3) further doubles the number of equivalent structural configurations, yielding *g*_+1*_ = 24 when also accounting for the spin degeneracy factor.

The consequences of degeneracy mismatches between ground and metastable defect structures can be exemplified by the temperature-dependent ‘reduced energy’ *E*_r_:^35–37^12
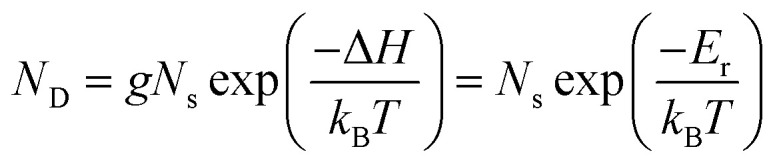

13 *E*_r_ = Δ*H* − *k*_B_*T* ln(*g*)

where *N*_D_ is the defect concentration, *g* is the site degeneracy, *N*_s_ is the concentration of lattice sites and Δ*H* is the defect formation enthalpy. The ‘reduced energies’ for Te_i_ are given in Table 1. The small reduced energy difference *E*_r_(300 K) = 18 meV between ground and metastable configurations for Te^+1^_i_ corresponds to an equilibrium concentration ratio of only 
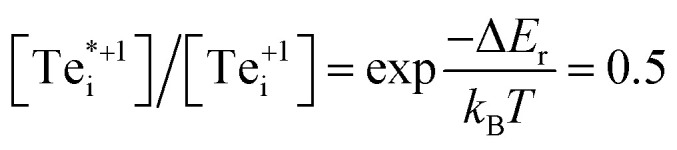
 at room temperature, while for Te^0^_i_ the greater degeneracy of the ground-state disfavours the metastable configuration. Notably, such entropic effects become even more significant at elevated temperatures (for example during crystal growth and annealing where defects are initially introduced—strongly affecting the resulting self-consistent Fermi level) and for cases with larger degeneracy differences (potentially over an order of magnitude). This phenomenon is another important consequence of symmetry-breaking and metastable configurations for point defects in semiconductors, which can significantly impact bulk defect concentrations and thus Fermi level position, in addition to the effects on non-radiative recombination discussed here.

**Table 1:** Point group symmetries, site, spin and total (configurational) degeneracies (*g*_site_, *g*_spin_ and *g*_total_), relative formation enthalpies (Δ*H*) and room temperature ‘reduced energies’ (*E*_r_(*T* = 300 K)) for tellurium interstitials 
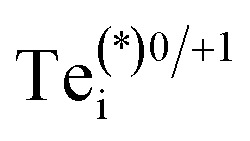
 in CdTe

Defect	Point symmetry	*g*_site_	*g*_spin_	*g*_total_	Δ*H* (meV)	*E*_r_(*T* = 300 K) (meV)
Te^0^_i_	*C*_2_	12	1	12	0	0
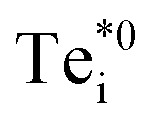	*C*_2v_	6	1	6	127	145
Te^+1^_i_	*C*_2v_	6	2	12	0	0
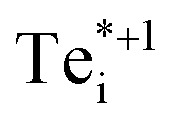	*C*_s_	12	2	24	36	18

#### Te_i_ in CdTe: charge capture

**Fig. 5:**
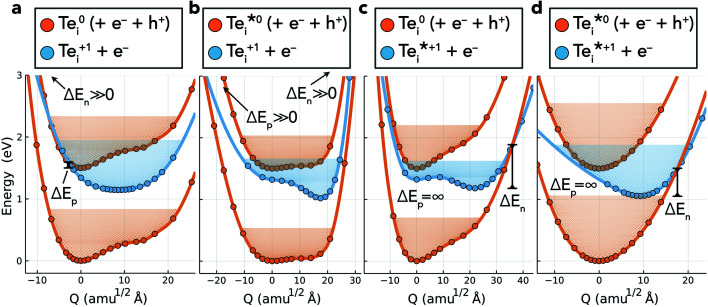
Potential energy surfaces (PESs) corresponding to the four Te_i_ (+^(^*^)^/0^(^*^)^) charge transition levels in CdTe. Filled circles denote datapoints calculated with hybrid DFT including spin–orbit coupling, and the solid lines are spline interpolations. The low-lying effective vibrational states are shown by the shaded regions, and Δ*E*_n/p_ represents the classical energy barrier to the capture transition. *Q* is the 1D structural coordinate along the path between equilibrium configurations, given in units of mass-weighted displacement. Transitions from the upper orange to blue PESs (Te^0^_i_ → Te^+1^_i_) correspond to hole capture, while those from blue to lower orange correspond to electron capture (Te^+1^_i_ → Te^0^_i_)

To determine the non-radiative electron/hole capture rates of the Te_i_ defect levels, we employed the one-dimensional configuration coordinate model of Alkauskas *et al.*^4^ as implemented in the CarrierCapture.jl package.^5^ The potential energy surfaces (PESs) for Te_i_ along the structural paths (configuration coordinates *Q*) between neutral and positive configurations are shown in Fig. 5.

**Fig. 6:**
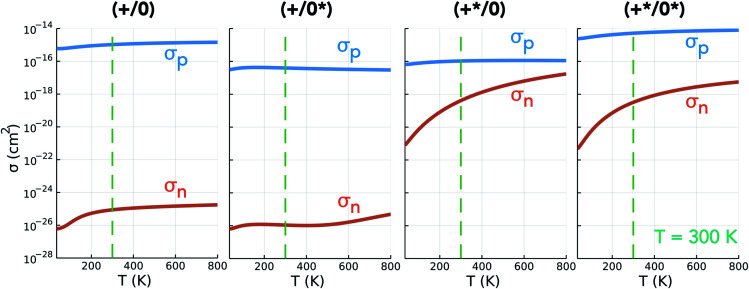
Electron and hole capture cross-section (*σ*_n_ and *σ*_p_) as functions of temperature *T* for the Te_i_ (+^(^*^)^/0^(^*^)^) charge transition levels in CdTe. Room temperature (*T* = 300 K) denoted by the dashed green line as a guide for typical solar cell operating conditions.

By calculating the electron–phonon coupling (*W*_if_ = 〈*Ψ*_i_|∂*Ĥ*/∂*Q*|*Ψ*_f_〉) and solving the 1D Schrödinger equation to determine the effective vibrational wavefunctions for the calculated energy surfaces, the non-radiative recombination activity of the corresponding defect transition levels (TLs) may be computed, with the results for Te_i_ provided in Table 2 and Fig. 6. The relationship between the calculated PESs and resultant capture rates can be intuitively understood through the classical energy barriers to charge capture, denoted by Δ*E*_n/p_ in Fig. 5. A small capture barrier Δ*E*_n/p_ and/or close overlap of the defect PESs near the equilibrium position typically yields fast carrier capture, while large barriers and/or significant separation of PESs results in slow capture rates.

**Table 2:** Transition level position (TL), mass-weighted displacement (Δ*Q*), classical capture barrier (Δ*E*), capture pathway degeneracy (*g*_capture_),^a^ electron–phonon coupling (*W*_if_), carrier capture coefficients (*C*_n/p_) and cross-sections *σ*_n/p_ for the Te_i_ (+^(^*^)^/0^(^*^)^) defect levels in CdTe, at temperature *T* = 300 K

TL (wrt. VBM) (eV)	Δ*Q* (amu^1/2^ Å)	Carrier	Δ*E* (eV)	*g*_capture_ a	*W*_if_ (eV amu^−1/2^ Å^−1^)	*C*_n/p_ (300 K) (cm^3^ s^−1^)	*σ*_n/p_ (300 K) (cm^2^)
(+/0) @ 0.35 eV	8.47	e^−^	>4	2	2.40 × 10^−2^	3.30 × 10^−18^	8.70 × 10^−26^
		h^+^	0.08	2	1.70 × 10^−2^	2.30 × 10^−8^	1.00 × 10^−15^
(+/0*) @ 0.48 eV	17.43	e^−^	2.23	12	2.40 × 10^−3^	4.10 × 10^−19^	1.10 × 10^−26^
		h^+^	3.43	8	1.30 × 10^−3^	8.60 × 10^−10^	4.00 × 10^−17^
(+*/0) @ 0.32 eV	21.94	e^−^	0.72	8	2.90 × 10^−3^	1.50 × 10^−11^	4.10 × 10^−19^
		h^+^	∞	2	5.70 × 10^−3^	2.30 × 10^−9^	1.10 × 10^−16^
(+*/0*) @ 0.44 eV	9.27	e^−^	0.43	1	2.10 × 10^−3^	1.20 × 10^−11^	3.20 × 10^−19^
		h^+^	∞	4	2.30 × 10^−2^	1.10 × 10^−7^	5.20 × 10^−15^

a The charge capture path degeneracy is the number of equivalent paths on the PES from the initial to final state, entering as a multiplicative factor in the capture rate.

Inspecting the defect PESs in Fig. 5 and corresponding capture rates in Table 2 & Fig. 6, we firstly note that all four potential transition pathways exhibit fast *hole* capture: 
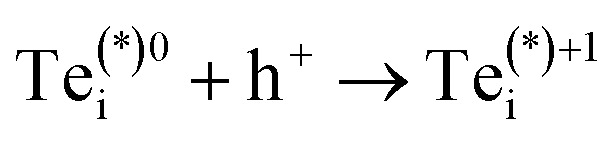
, with close overlap of the PESs about the upper orange 
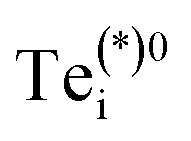
 minimum in each case. This behaviour is consistent with the conventional rationale of fast hole capture for defect levels located near the VBM, as is the case here (Fig. 4). The large hole capture cross-sections *σ*_p_ ∼ 10^−15^ cm^2^ – of similar magnitude to its atomic cross-section *σ*_Te_ = π(*a*_Te_)^2^ ≃ 1.5 × 10^−15^ cm^2^ – classify Te_i_ as a ‘giant’ hole trap.^25,38,39^ On the other hand, we witness negligible *electron* capture due to high barriers and low PES overlap for the (+/0) and (+/0*) transition levels (Fig. 5a and b), with tiny capture cross-sections *σ*_n_ ∼ 10^−26^ cm^2^. It is only when 
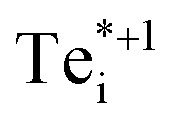
 is involved, as in the (+*/0) and (+*/0*) levels in Fig. 5c and d, that a tractable electron capture barrier Δ*E*_n_ emerges, yielding an increase in the electron capture rate by over 6 orders of magnitude to *σ*_n_ ∼ 10^−19^ cm^2^ (Table 2 and Fig. 6). To contextualise, we note that ‘giant’ traps or ‘killer’ defects are typically classified as those with capture cross-sections *σ* ∼ 10^−15^ to 10^−13^ cm^2^, for moderate traps *σ* ∼ 10^−20^ to 10^−15^ cm^2^ and weak traps *σ* < 10^−20^ cm^2^.^4,25,39^ Of course, the overall rate of capture (*R*) remains dependent on the defect and carrier concentrations (*N*_D_ and *n*) in the material (*R* ∝ *N*_D_*σ*_n_), meaning defects with moderate capture cross-sections can act as performance-limiting species if they form easily in the bulk, as is the case for Te_i_ in commercial Te-rich CdTe.

The slow electron capture rates of the (+/0) and (+/0*) TLs rule out the possibility of significant Shockley–Read–Hall e–h recombination through the typical two-step process (Fig. 1b), or indeed through the recently reported capture–relaxation–capture process discussed previously (Fig. 1c). The large *σ*_n_ of 
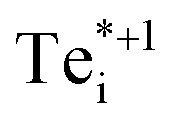
 does, however, permit electron–hole recombination to proceed through the three-step and four-step process shown in Fig. 1d and e, involving internal excitation from the ground-state of the positive interstitial 
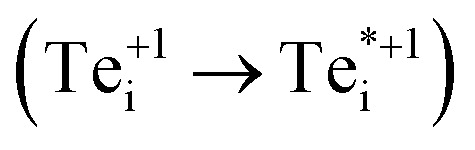
, before electron capture to either the ground-state or metastable neutral interstitial 
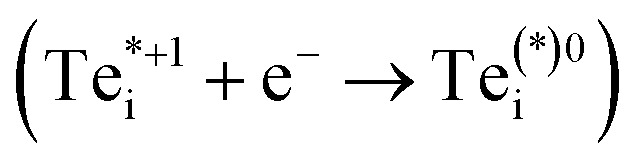
, and finally fast hole capture to complete the recombination cycle. For the thermal excitation 
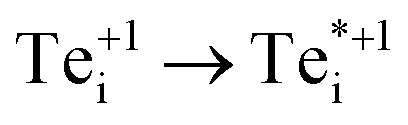
, we calculate an energy barrier of 0.08 eV (Fig. S2†) and an effective vibrational attempt frequency *ν* = 0.40 THz, yielding a room temperature transition rate *k*_+/+*_ = 7.6 × 10^10^ s^−1^, using transition state theory. This internal conversion occurs far more rapidly than the rate-limiting electron capture process (
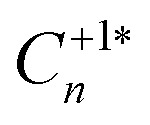
*n* ≃ (1.5 × 10^−11^ cm^3^ s^−1^)(10^12^ cm^−3^) ≃ 10^1^ s^−1^), as is also the case for the barrier-less 
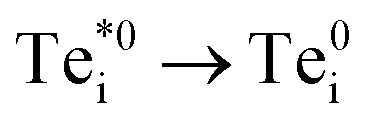
 vibrational relaxation. Further details for these calculations are provided in Section S3.†

**Fig. 7:**
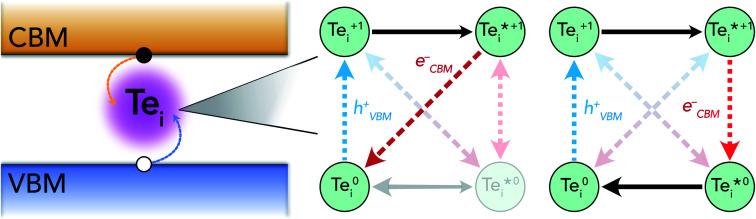
Schematic of the proposed non-radiative recombination mechanism at tellurium interstitials in CdTe. Red/blue arrows denote electron/hole capture, and black arrows indicate internal conversion reactions.

We observe facile thermal transformation between Te_i_ defect structures under typical solar cell operating conditions (black arrows in Fig. 1d, e and 7), with electron capture by 
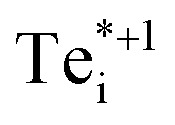
 representing the rate-limiting step in the recombination kinetics—now proceeding >6 orders of magnitude faster than for the ground-state Te^+1^_i_. In summary, without the presence of the metastable 
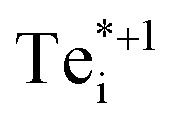
, tellurium interstitials would behave as benign defect centres, only capable of capturing holes and later emitting them, reducing carrier mobilities but having no effect on recombination. However, by introducing alternative pathways for electron capture to proceed, the low-lying metastable +1 structure facilitates the 3-step and 4-step recombination cycles shown in Fig. 1d, e and 7, transforming Te_i_ into an important recombination centre in Te-rich CdTe.

#### Capture *from* metastable defects: general considerations

As before, for *ε*(q*/*q* ± 1) to enter the recombination cycle, it must be positioned within the band gap, setting an upper limit to the relative energy of the metastable structure D*^q^ (eqn (S25) and (S26)†). The higher energy of the metastable defect (D*^q^), now the initial state in the capture process, results in a charge transition level *ε*(q*/*q* ± 1) which is now *further* from the corresponding band edge than *ε*(*q*/*q* ± 1) (Fig. 2, 4 & S1, and eqn (S21) & (S22)†). While a deeper defect level would typically imply slower capture velocities under the classic recombination model,^1,9,19,40^ the complexity of defect PESs results in myriad situations where this conventional wisdom no longer holds. Indeed, we witness here that the (+*/0) charge transition level, located *furthest* from the CBM of all Te_i_ (+^(^*^)^/0^(^*^)^) defect levels (Fig. 4), exhibits the greatest rate of electron capture (Table 2 and Fig. 6). This unintuitive behaviour can be attributed to the significant structural distortion and anharmonicity along the path between these defects (Fig. 5), highlighting the major impact that symmetry-breaking can have on the properties of defects in semiconductors.^11^

Accordingly, these findings yield important considerations. Semiconductors exhibiting strong anharmonicity and mixed ionic–covalent bonding (*e.g.* Bi, Sb and Sn-based materials),^1,41^ low crystal symmetry (*e.g.* Sb_2_X_3_)^42,43^ and/or compositional complexity (*e.g.* multinary compounds such as CZTS and double perovskites)^44–46^ are likely to manifest local minima on defect energy surfaces. Low-energy metastable defect structures may be especially prevalent in emerging inorganic PV materials which tend to exhibit these properties, highlighting the potential importance of these species to investigations of non-radiative recombination and open-circuit voltage deficits in emerging solar cells. Indeed, even in the case of the high-symmetry binary semiconductor CdTe, many low energy metastable structures exist, introducing 18 additional charge transition levels in the gap and accelerating the carrier trapping at Te_i_ and V_Cd_.^9^ An important factor in the emergence of the thermal-excitation capture pathway here is the high mobility of the Te interstitial in CdTe, as demonstrated by the low diffusion barriers calculated in this and previous works.^26,33,34^ The facile ionic diffusion of Te_i_ is a simultaneous consequence of the soft, anharmonic PESs which cause the ready accessibility of distinct structural motifs (*i.e.* low-lying metastable structures), facilitating this complex recombination pathway. Thus, we additionally propose rapid diffusion as a potential indicator of this behaviour at other semiconductor defect centres. Finally, we note that the very presence of metastable structures on the defect PES implies a softer and more anharmonic energy landscape, with smaller barriers between structures. As exemplified in this and other studies,^7,9,21,47^ such effects often yield unintuitive recombination kinetics, with fast trapping despite deep energy levels.

In conclusion, metastable defect structures impact non-radiative recombination in semiconductors. They introduce a complex set of potential recombination paths at defects, akin to chemical reaction mechanisms, with the overall kinetics being a function of the individual transition rates. In addition to the thermal-relaxation pathway discussed in recent works,^9,16–18^ we demonstrate that metastable defect structures can also enter the recombination cycle *via* thermal excitation, provided the internal transformation energy barrier is sufficiently small. We focus on the tellurium interstitial (Te_i_) in CdTe solar cells as an illustrative example of this phenomenon, exhibiting complex 3-step and 4-step recombination cycles which, to our knowledge, have not been previously reported in the literature. In addition to demonstrating the major potential impact of metastable structures on defect-mediated electron–hole recombination, Te_i_ serves a clear example of anharmonicity causing deviation from the typical trend of reduced capture rates with deeper defect levels (*i.e.* greater energy separation from the band edge). Finally, we highlight implications to the broader field of photovoltaic materials research. We pose that metastable defect structures are more important to non-radiative recombination than currently understood, particularly in the case of emerging inorganic PV materials which often exhibit reduced crystal symmetries, mixed ionic–covalent bonding, multinary compositions and/or highly-mobile defects like Te_i_, where anharmonicity, symmetry-breaking and soft potential energy surfaces are likely to yield many low-lying, easily-accessible metastable structures, enabling the behaviour discussed here.

## Methods

Anharmonic carrier capture coefficients were calculated using CarrierCapture.jl,^5^ with electron–phonon coupling matrix elements determined using the method outlined in ref. 6. In-depth details of the computational implementation are provided in ref. 9. All of the underlying total energy calculations were performed using Density Functional Theory (DFT) within periodic boundary conditions through the Vienna *Ab Initio* Simulation Package (VASP), employing Projector-Augmented Wave (PAW) pseudopotentials.^48–51^ The screened hybrid DFT exchange–correlation functional of Heyd, Scuseria and Ernzerhof (HSE),^52^ including spin–orbit interactions (SOC), was used for all calculations. An exact Hartree–Fock exchange fraction of *α*_exx_ = 34.5% was employed in the hybrid DFT model, reproducing the room temperature experimental band gap of 1.5 eV for CdTe. A 64-atom, 13.1 Å cubic supercell was used for defect calculations, with a well-converged 450 eV plane-wave energy cutoff, a 2 × 2 × 2 *Γ*-centred Monkhorst–Pack ***k***-point mesh and a force convergence criterion of 0.01 eV Å^−1^ for geometry optimization. The ShakeNBreak defect structure-searching method which aids the efficient location of ground and metastable structures for defects in solids was employed.^11,12^

## Data availability

Data produced during this work is freely available at https://doi.org/10.5281/zenodo.5999057.

## Conflicts of interest

There are no conflicts to declare.

## Supplementary Material

FD-239-D2FD00043A-s001
