# Supplementary material for: Impact of metastable defect structures on carrier recombination in solar cells
Source: Faraday Discuss. 2022 Apr 11;239:339–56. doi: 10.1039/d2fd00043a (PMC9615105; doi:10.1039/d2fd00043a)
Supplement: FD-239-D2FD00043A-s001 [file FD-239-D2FD00043A-s001.pdf]

Electronic Supplementary Material (ESI) for Faraday Discussions.
This journal is © The Royal Society of Chemistry 2022

Supplementary Material: Impact of metastable defect structures on carrier recombination in solar cells

S1 Charge Transition Levels Involving Metastable Defects

The formation energy $\Delta H_{D,q}(E_F,\mu)$ of a defect D in charge state q, calculated using the conventional supercell method, is given by:[1,2]

$$\Delta H_{D,q}(E_F,\mu) \;=\; [E_{D,q} \;-\; E_H] \;-\; \sum_i n_i\mu_i \;+\; qE_F \;+\; E_{Corr}(q) \tag{S1}$$

where $E_{D,q}$ is the calculated energy of the defect supercell and E_H is the energy of an equivalent *pristine* host supercell. The second term ($-\sum_i n_i\mu_i$) accounts for the thermodynamic cost of exchanging n_i atoms with their reservoir chemical potential(s) μ_i, to form the defect D_q from the ideal bulk material. Similarly, qE_F represents the electron chemical potential contribution, while $E_{Corr}(q)$ is a correction for any finite-size supercell effects.

Defect thermodynamic charge transition levels $\varepsilon(q_1/q_2)$ are defined as the Fermi level position for which the formation energies of the q_1 and q_2 charge defects are equal:[2]

$$\varepsilon(q_1/q_2) = E_F; \;\; \Delta H_{D,q_1}(E_F) = \Delta H_{D,q_2}(E_F) \tag{S2}$$

For a Fermi level position above this defect transition level, the more negative charge state will be favoured, whereas the positive state will be favoured for a lower lying Fermi level. Due to the charge-dependent relationship between defect formation energy and Fermi level energy ($\Delta H_{D,q}(E_F) \propto qE_F$; Eq. (S1)), an expression for the charge transition level $\varepsilon(q_1/q_2)$ can be derived following:

$$\Delta H_{D,q_1} = \Delta H_{D,q_1}(E_F = 0) + q_1E_F; \;\; \Delta H_{D,q_2} = \Delta H_{D,q_2}(E_F = 0) + q_2E_F \tag{S3}$$

Setting $\Delta H_{D,q_1}$ equal to $\Delta H_{D,q_2}$ to solve for E_F (i.e. the Fermi level position for which the defect formation energies are equal $:= \varepsilon(q_1/q_2)$):

$$\Delta H_{D,q_1}(E_F = 0) + q_1E_F = \Delta H_{D,q_2}(E_F = 0) + q_2E_F \tag{S4}$$

$$\Delta H_{D,q_1}(E_F = 0) - \Delta H_{D,q_2}(E_F = 0) = (q_2 - q_1)E_F \tag{S5}$$

$$\longrightarrow E_F := \varepsilon(q_1/q_2) = \frac{\Delta H_{D,q_1}(E_F = 0) - \Delta H_{D,q_2}(E_F = 0)}{q_2 - q_1} \tag{S6}$$

where $\Delta H_{D,q}(E_F = 0)$ is the formation energy of defect D with charge q (defined in Eq. (S1)), when the Fermi level is at the zero-reference point (the VBM, by convention).

The shifts in charge transition level positions when metastable defect structures are involved are derived in Sections S1.1 to S1.3 and illustrated in Fig. S1.

S1.1 Charge Capture *Into* Metastable Defects; $D^q \rightarrow D^{*q\pm1}$

Taking $q_1 = q$ and $q_2 = q \pm 1$ (as charge capture is a single-carrier process) and denoting the energy of the metastable defect state $\Delta H_{D^*,q\pm1}$ as:

$$\Delta H_{D^*,q\pm1} = \Delta H_{D,q\pm1} + \Delta E \tag{S7}$$

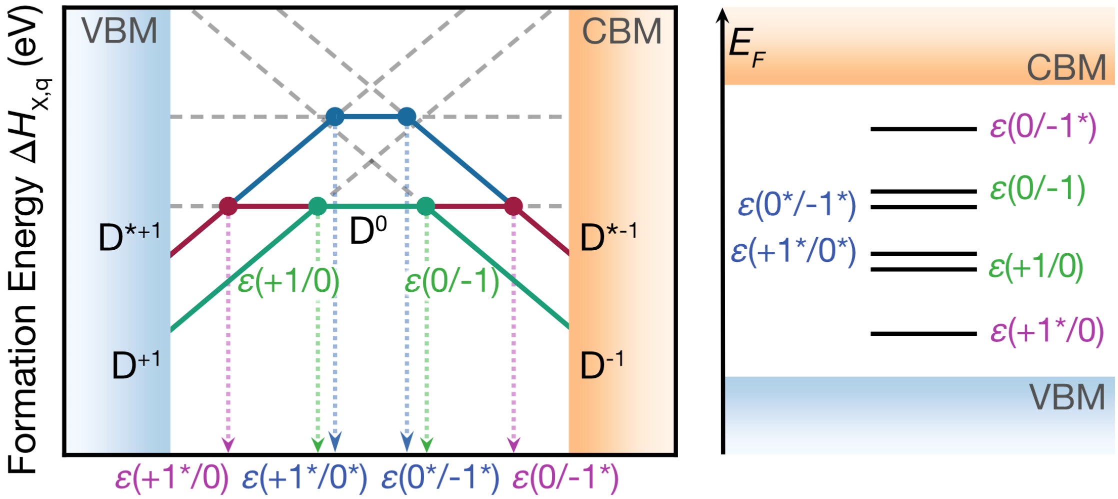

Fig. S1 **(Left)** Defect formation energy diagram showing the positions of charge transition levels $\varepsilon(q^{(*)}/q^{(*)} \pm 1)$ involving zero (green), one (magenta) or two (blue) metastable defect structures. **(Right)** Vertical energy band diagram showing the transition level positions within the bandgap.

where ΔE is the energy of the metastable defect relative to the ground-state structure, the charge transition level $\varepsilon(q_1/q_2^*)$ can thus be written:

$$\varepsilon(q/q^* \pm 1) = \frac{\Delta H_{D,q}(E_F = 0) - \Delta H_{D^*,q\pm 1}(E_F = 0)}{q \pm 1 - q} = \frac{\Delta H_{D,q}(E_F = 0) - (\Delta H_{D,q\pm 1}(E_F = 0) + \Delta E)}{q \pm 1 - q} \tag{S8}$$

$$\varepsilon(q/q^* \pm 1) = \frac{\Delta H_{D,q}(E_F = 0) - \Delta H_{D,q\pm 1}(E_F = 0)}{q \pm 1 - q} - \frac{\Delta E}{q \pm 1 - q} \tag{S9}$$

$$\varepsilon(q/q^* \pm 1) = \varepsilon(q/q \pm 1) - \frac{\Delta E}{\pm 1} \tag{S10}$$

$$\varepsilon(q/q^* \pm 1) = \varepsilon(q/q \pm 1) \mp \Delta E \tag{S11}$$

$$e^- \text{ capture} \implies \varepsilon(q/q^* - 1) = \varepsilon(q/q - 1) + \Delta E \tag{S12}$$

$$h^+ \text{ capture} \implies \varepsilon(q/q^* + 1) = \varepsilon(q/q + 1) - \Delta E \tag{S13}$$

Thus we witness that for charge capture *into* a metastable structure, $D^q \rightarrow D^{*q\pm 1}$, the transition level will move an energy ΔE *closer to* the corresponding band-edge (i.e. to higher energy for electron capture or to lower energy for hole capture), assuming a transition level $\varepsilon(q/q \pm 1)$ initially located within the bandgap.

Given the requirement that $\varepsilon(q/q^* \pm 1)$ must lie within the bandgap in order to effectuate non-radiative recombination, we have the constraint:

$$0 < \varepsilon(q/q^* \pm 1) < E_g \tag{S14}$$

$$0 < \varepsilon(q/q \pm 1) \mp \Delta E < E_g \tag{S15}$$

Thus for electron capture we have:

$$\varepsilon(q/q - 1) + \Delta E < E_g \tag{S16}$$

$$\longrightarrow \Delta E < E_g - \varepsilon(q/q - 1) \tag{S17}$$

and for hole capture:

$$0 < \varepsilon(q/q + 1) - \Delta E \tag{S18}$$

$$\longrightarrow \Delta E < \varepsilon(q/q + 1) \tag{S19}$$

Therefore, we see that the energetic separation between the ground-state transition level $\varepsilon(q/q \pm 1)$ and the

This journal is © The Royal Society of Chemistry 2022

corresponding band-edge sets the energy window within which the metastable structure must lie in order to affect the electron-hole recombination process (as demonstrated in Fig. S1).

S1.2 Charge Capture *From* Metastable Defects; $D^{*q} \rightarrow D^{q\pm1}$

In the case of charge capture *from* metastable structures, the same equations from the previous section hold, just with a reversal of the sign of the ΔE term:

$$\varepsilon(q^*/q \pm 1) = \varepsilon(q/q \pm 1) \pm \Delta E \tag{S20}$$

$$e^- \text{ capture} \implies \varepsilon(q^*/q-1) = \varepsilon(q/q-1) - \Delta E \tag{S21}$$

$$h^+ \text{ capture} \implies \varepsilon(q^*/q+1) = \varepsilon(q/q+1) + \Delta E \tag{S22}$$

Hence for charge capture *from* a metastable structure, $D^{*q} \rightarrow D^{q\pm1}$, the transition level will move an energy ΔE *further from* the corresponding band-edge, assuming a transition level $\varepsilon(q/q \pm 1)$ initially located within the bandgap.

Likewise, we obtain the following reversed constraints on the relative energy of the metastable structure, to impact carrier recombination:

$$0 < \varepsilon(q^*/q \pm 1) < E_g \tag{S23}$$

$$0 < \varepsilon(q/q \pm 1) \pm \Delta E < E_g \tag{S24}$$

$$e^- \text{ capture} \implies \Delta E < \varepsilon(q/q-1) \tag{S25}$$

$$h^+ \text{ capture} \implies \Delta E < E_g - \varepsilon(q/q+1) \tag{S26}$$

S1.3 Charge Capture Between Two Metastable Defect Structures; $D^{*q} \longleftrightarrow D^{*q\pm1}$

As demonstrated in Fig. S1, the positions of defect charge transition levels involving metastable configurations for both charge states are dictated by the balance of the relative energies of both metastable structures $D^{*q(\pm1)}$ with respect to their ground-state counterparts $D^{q(\pm1)}$, such that:

$$\varepsilon(q^*/q^* \pm 1) = \varepsilon(q/q \pm 1) \pm \Delta E_q \mp \Delta E_{q\pm1} \tag{S27}$$

with the same constraint that $\varepsilon(q^*/q^* \pm 1)$ must lie within the bandgap in order for recombination to be energetically permitted.

S2 Te_i in CdTe: Nudged Elastic Band (NEB) Calculations

Fig. S2 shows the calculated minimum energy paths between the groundstate and metastable structures for ${Te_i}^0$ and ${Te_i}^{+1}$. As mentioned in the main text, in both cases the metastable structures represent the intermediate state in the diffusion mechanism of Te_i in CdTe. Thus the energy barriers to structural transition also represent the activation energies for interstitial diffusion of tellurium in CdTe.

Using the HSE(34.5%)+SOC hybrid DFT functional, we calculate transition energy barriers of 0.13 eV and 0.08 eV respectively for ${Te_i}^0 \rightarrow {Te_i}^{*0}$ and ${Te_i}^{+1} \rightarrow {Te_i}^{*+1}$. We note that Ma *et al.*, Yang *et al.*[4,5] both found similar barriers of 0.09 eV for the ${Te_i}^0 \rightarrow {Te_i}^{*0}$ transition, with semi-local (GGA) and hybrid (HSE06) DFT respectively, and no local stability about ${Te_i}^{*0}$. On the other hand, Selvaraj *et al.*[6] calculated a barrier of 0.24 eV using local (LDA) DFT, with a 0.03 eV window of stability for the intermediate state.

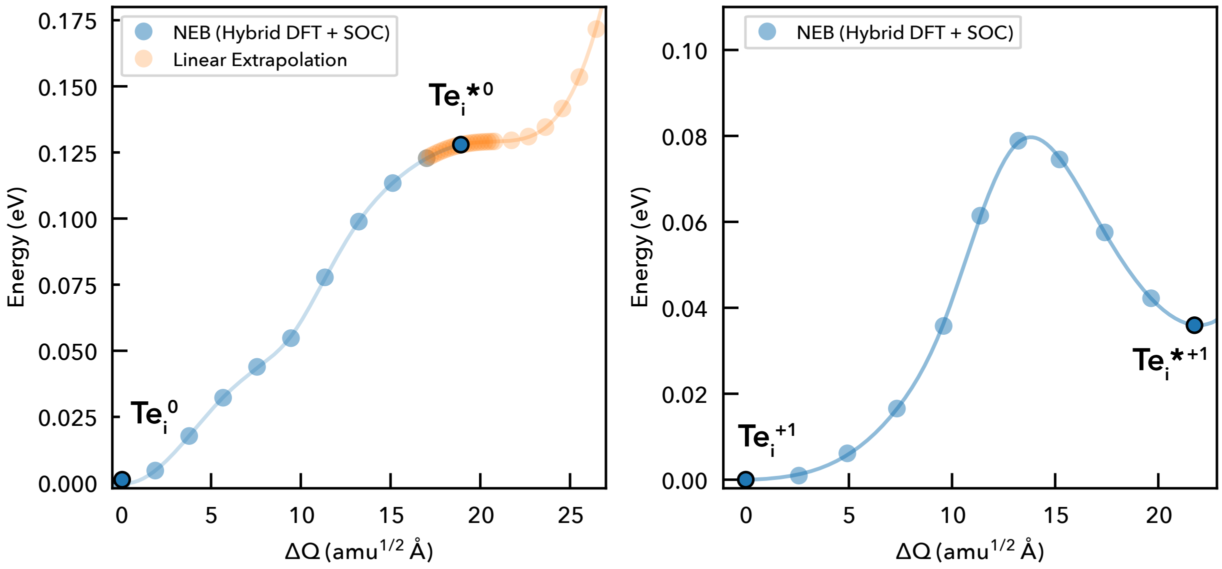

Fig. S2 Minimum energy paths along the potential energy surface between groundstate and metastable structures for **(left)** Te_i^0 and **(right)** Te_i^{+1}, calculated using the Nudged Elastic Band (NEB) method.[3] Filled circles represent calculated data points and the solid line is a spline fit. X-axis given in units of mass-weighted displacement.

S3 Te_i in CdTe: Transition State Theory

To estimate the rate of internal structural transformation for point defects, we can invoke Transition State Theory,[7] which gives the rate of reaction k as:

$$k = \nu g \exp(-\frac{\Delta E}{k_B T}) \tag{S28}$$

where ν is the effective (vibrational) attempt frequency, g is the ratio of the degeneracies of the final and initial states and ΔE is the activation energy barrier.

Using the calculated energy barrier of 0.08 eV and effective vibrational attempt frequency $\nu = 0.40$ THz (from parabolic fitting of the Te_i^{+1} local minimum shown in Fig. S2), the $Te_i^{+1} \rightarrow Te_i^{*+1}$ transition is estimated to proceed at a rate:

$$k_{+/+^*} = (4 \times 10^{11})(4)\exp(-\frac{0.08}{(0.0259)(300)}) = 7.6 \times 10^{10}\,\mathrm{s}^{-1} \quad @\ \mathrm{T} = 300\,\mathrm{K}$$

S3.1 Internal Conversion vs Charge Capture Rates

To estimate the transition barrier height at which internal conversion and charge capture would have comparable speeds, we equate their reaction rates:

$$k = \nu g \exp(-\frac{\Delta E}{k_B T}) = \sigma_n v_{th} n \tag{S29}$$

where σ_n is the capture cross-section, v_{th} is the carrier thermal velocity and n is the minority carrier concentration (minority carrier capture often represents the rate-limiting step in the electron-hole recombination cycle). This is then rearranged to:

$$\exp(-\frac{\Delta E}{k_B T}) = \frac{\sigma_n v_{th} n}{\nu g} \tag{S30}$$

$$\Delta E = -k_B T \ln \frac{\sigma_n v_{th} n}{\nu g} \tag{S31}$$

This journal is © The Royal Society of Chemistry 2022

Typical minority carrier concentrations are $n \sim 1 \times 10^{14}\,\mathrm{cm}^{-3}$, with velocities $v_{th} \sim 1 \times 10^{7}\,\mathrm{cm/s}$. Taking these values along with the ranges $\sigma \sim 10^{-15} - 10^{-13}\,\mathrm{cm}^2$ for 'killer' defect centres[8–10] and usual attempt frequencies of $\nu \sim 0.5-10\,\mathrm{THz}$,[5,11,12] we obtain the following upper and lower estimates for ΔE:

$$\Delta E_{max} \; = \; -(0.0259)\ln\frac{(10^{-15})\,(10^{7})\,(10^{14})}{(10^{13})\,(1)} \; = \; 0.42\,\mathrm{eV} \;\; @\ \mathrm{T} = 300\,\mathrm{K} \tag{S32}$$

$$\Delta E_{min} \; = \; -(0.0259)\ln\frac{(10^{-13})\,(10^{7})\,(10^{14})}{(5\times 10^{11})\,(1)} \; = \; 0.22\,\mathrm{eV} \;\; @\ \mathrm{T} = 300\,\mathrm{K} \tag{S33}$$

Thus internal conversion is likely to be the rate-limiting step in the recombination cycle of a fast-capturing defect if the transition energy barrier ΔE is greater than $0.4\,\mathrm{eV}$, while barriers less than $0.2\,\mathrm{eV}$ should yield structural conversion rates far quicker than charge capture. For barriers between these extremal limits, which process becomes the rate-limiting step will depend on the specific parameters for that system.

Supplemental References

1 Y.-T. Huang, S. R. Kavanagh, D. O. Scanlon, A. Walsh and R. L. Z. Hoye, *Nanotechnology*, 2021, **32**, 132004.

2 C. Freysoldt, B. Grabowski, T. Hickel, J. Neugebauer, G. Kresse, A. Janotti and C. G. Van de Walle, *Reviews of Modern Physics*, 2014, **86**, 253–305.

3 H. Jónsson, G. Mills and K. W. Jacobsen, *Classical and Quantum Dynamics in Condensed Phase Simulations*, World Scientific, 1998, pp. 385–404.

4 J. Ma, J. Yang, S.-H. Wei and J. L. F. Da Silva, *Physical Review B*, 2014, **90**, 155208.

5 J.-H. Yang, J.-S. Park, J. Kang and S.-H. Wei, *Physical Review B*, 2015, **91**, 075202.

6 S. C. Selvaraj, S. Gupta, D. Caliste and P. Pochet, *Applied Physics Letters*, 2021, **119**, 062105.

7 G. H. Vineyard, *Journal of Physics and Chemistry of Solids*, 1957, **3**, 121–127.

8 A. M. Stoneham, *Theory of Defects in Solids: Electronic Structure of Defects in Insulators and Semiconductors*, Oxford University Press, 2001.

9 A. Alkauskas, Q. Yan and C. G. Van de Walle, *Physical Review B*, 2014, **90**, 075202.

10 S. Kim, J. A. Márquez, T. Unold and A. Walsh, *Energy & Environmental Science*, 2020, 1481–1491.

11 S. R. Kavanagh, A. Walsh and D. O. Scanlon, *ACS Energy Letters*, 2021, **6**, 1392–1398.

12 J.-H. Yang, L. Shi, L.-W. Wang and S.-H. Wei, *Scientific Reports*, 2016, **6**, 21712.
